# Temperature Affects Musculoskeletal Development and Muscle Lipid Metabolism of Gilthead Sea Bream (*Sparus aurata*)

**DOI:** 10.3389/fendo.2019.00173

**Published:** 2019-03-22

**Authors:** Sara Balbuena-Pecino, Natàlia Riera-Heredia, Emilio J. Vélez, Joaquim Gutiérrez, Isabel Navarro, Miquel Riera-Codina, Encarnación Capilla

**Affiliations:** Departament de Biologia Cel·lular, Fisiologia i Immunologia, Facultat de Biologia, Universitat de Barcelona, Barcelona, Spain

**Keywords:** thermal exposure, bone cells, white muscle, mineralization, GH/IGFs system, lipid catabolism

## Abstract

World population is expected to increase to approximately 9 thousand million people by 2050 with a consequent food security decline. Besides, climate change is a major challenge that humanity is facing, with a predicted rise in mean sea surface temperature of more than 2°C during this century. This study aims to determine whether a rearing temperature of 19, 24, or 28°C may influence musculoskeletal development and muscle lipid metabolism in gilthead sea bream juveniles. The expression of growth hormone (GH)/insulin-like growth factors (IGFs) system-, osteogenic-, myogenic-, and lipid metabolism-related genes in bone and/or white muscle of treated fish, and the *in vitro* viability, mineralization, and osteogenic genes expression in primary cultured cells derived from bone of the same fish were analyzed. The highest temperature significantly down-regulated *igf-1, igf-2*, the receptor *igf-1ra*, and the binding proteins *igfbp-4* and *igfbp-5b* in bone, and in muscle, *igf-1* and *igf-1ra*, suggesting impaired musculoskeletal development. Concerning myogenic factors expression, contrary responses were observed, since the increase to 24°C significantly down-regulated *myod1* and *mrf4*, while at 28°C *myod2* and *myogenin* were significantly up-regulated. Moreover, in the muscle tissue, the expression of the fatty acid transporters *cd36* and *fabp11*, and the lipases *lipa* and *lpl-lk* resulted significantly increased at elevated temperatures, whereas β-oxidation markers *cpt1a* and *cpt1b* were significantly reduced. Regarding the primary cultured bone-derived cells, a significant up-regulation of the extracellular matrix proteins *on, op*, and *ocn* expression was found with increased temperatures, together with a gradual decrease in mineralization along with fish rearing temperature. Overall, these results suggest that increasing water temperature in this species appears to induce unfavorable growth and development of bone and muscle, through modulating the expression of different members of the GH/IGFs axis, myogenic and osteogenic genes, while accelerating the utilization of lipids as an energy source, although less efficiently than at optimal temperatures.

## Introduction

Nowadays, society is facing one of the greatest world challenges: how to feed 9 thousand million people by 2050 in the context of global change and economic and financial uncertainty (1, 2). In this situation, aquaculture has a relevant role, satisfying the growing need of fish, and gilthead sea bream (*Sparus aurata*, L.) has become over the last 30 years one of the most important fish species farmed in the Mediterranean area (3). Besides food insecurity, climate change is also a major global challenge that concerns humanity. Warming of the climate system is unequivocal and particularly relevant for this study, the mean sea surface temperature may increase more than 2°C by the end of this century (4). Increased water temperature is known to directly influence several biochemical and physiological processes in ectothermic fish (5), including growth or metabolic rate (6).

Temperature can influence the growth hormone (GH)/insulin-like growth factors (IGFs) system, the main endocrine axis controlling growth in vertebrates. Previous studies have reported a link between environmental temperature and plasma levels of IGF-1 and GH, independently of the nutritional status (7, 8). The GH/IGFs system includes peptides (IGF-1 and IGF-2), IGF-1 receptors (IGF-1Ra and IGF-1Rb), as well as is composed of six IGF binding proteins (IGFBPs) that can exert different effects on IGFs function depending on the cellular context (9, 10). Interestingly, IGFBP-2 in teleosts is the main circulatory binding protein and shows a physiological regulation similar to the most relevant one in mammals, IGFBP-3 (11). Furthermore, elevated temperatures, along with other risk factors such as mineral and vitamin deficiencies, light regimes or fast growth, have been linked in fish with increased occurrence of skeletal anomalies (12). In fact, fast muscle growth can exert high mechanical pressure on the developing bone, hence, synchronicity between bone and muscle growth is required for proper musculoskeletal development [reviewed by Ytteborg et al. (13)]. The higher prevalence of vertebral deformities as a result of increased temperature during the early stages of development has been documented in gilthead sea bream (14), as well as in other species including *Solea senegalensis* (15), *Salmo salar* (16), or *Dicentrarchus labrax* (17). In the case of Sparids, the presence of abnormalities is more evident in larvae reared below 15°C and above 22°C (18), and recent studies have demonstrated in this species that thermal imprinting during embryogenesis causes long-term effects on bone physiology (19, 20). In this sense, the increase in temperature can be recognized as one important problem for aquaculture and animal welfare in a global climate change context.

Cellular and molecular mechanisms for musculoskeletal development in teleost fish have been demonstrated to be similar than in mammals. Osteoblasts, as well as myocytes, arise from mesenchymal stem cells (MSCs), precursor cells that are also able to differentiate into other cell types like chondroblasts or adipocytes after the coordinated induction of key transcription factors expression. Recently, morphological and molecular characterization of a bone-derived cell culture of gilthead sea bream has been reported (20, 21), and the ability of those MSCs to differentiate into other cell types such as adipocyte-like cells has been demonstrated (22). However, these multipotent cells have not been deeply characterized at a structural/functional level. Concerning the main regulators of bone development at a transcriptional level, Runt-related transcription factor 2 (Runx2) is the one required for commitment toward the osteogenic lineage. Afterwards, osteoblasts express molecules of the extracellular matrix (ECM), which include structural fibers as collagen or fibronectin but also non-collagenous proteins that regulate mineralization of the ECM such as osteonectin (ON), osteopontin (OP), and osteocalcin (OCN) (20). In the case of muscle, abundant studies using a satellite cell model system have properly characterized the process of myogenesis in gilthead sea bream (23). The coordinated expression of myogenic regulatory factors (MRFs) is also required for myogenesis to properly occur. Among these transcription factors, Myf5 and MyoD are involved in myocytes activation and proliferation, whereas Myogenin and MRF4 act later allowing myotube formation and maturation (24).

In addition to growth, increased water temperature is also known to directly affect energy demand in ectotherms, and consequently to exert an impact in lipid metabolism and the use of fat depots (25). Lipids are an important energy source for fish skeletal muscle. Lipases such as lipoprotein lipase (LPL) and lipase A (LIPA) can provide fatty acids from triglycerides circulating in the form of chylomicrons and very low-density lipoproteins. Then, fatty acid transporters such as CD36 and FATP1, which are nutritionally and hormonally regulated in fish muscle (26, 27), facilitate the entry of these fatty acids into the cell. Endogenous stored triglycerides, when necessary, can also be hydrolyzed by other lipases as the hormone-sensitive lipase (HSL). Then, non-esterified fatty acids undergo β-oxidation in the mitochondrial matrix (28).

In this framework, the aim of the present study was to evaluate the effects of three increasing rearing temperatures (19, 24, and 28°C) in gilthead sea bream juveniles through an *in vivo*/*in vitro* approach. First, the *in vivo* expression of GH/IGFs axis-, osteogenic-, myogenic- and lipid metabolism-related genes in bone and/or white muscle was determined, and then, the *in vitro* development and expression of osteogenic genes in primary cultured bone-derived cells. All this performed to extend the knowledge of the possible impacts of global climate change on musculoskeletal growth and the physiology in this important aquaculture marine species.

## Materials and Methods

### Animals and Experimental Trial

**Figure 1:**
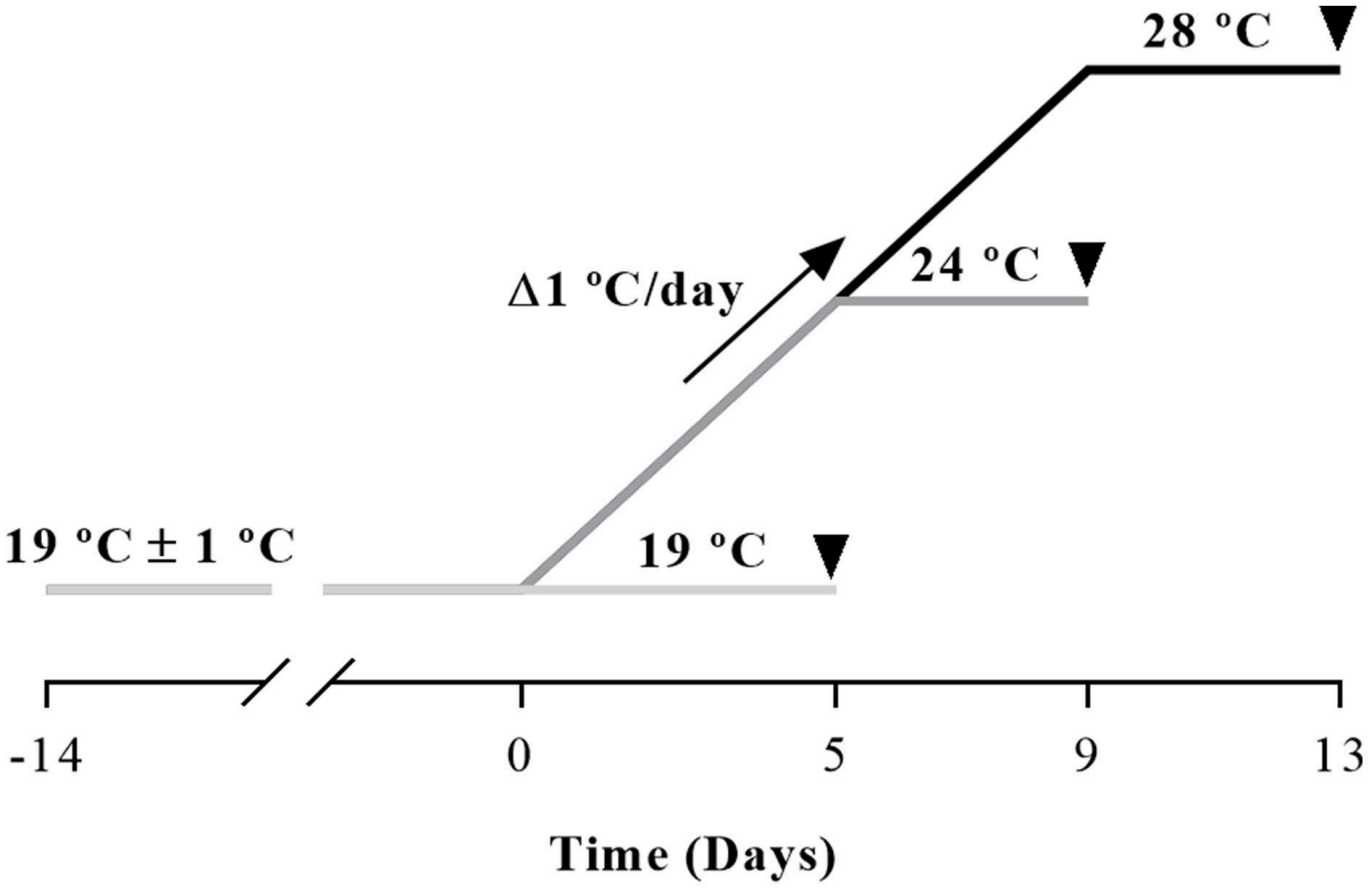
Schematic design of the experimental trial. Gilthead sea bream juveniles were maintained 2 weeks at room temperature (19 ± 1°C) for acclimation before temperature was raised at a rate of 1°C/day with a 250 W thermostatic heater until achieving the desired 24 or 28°C. Fish were held for 3 days at each corresponding temperature and were sampled on the fourth day as indicated by the arrowheads.

Gilthead sea bream juveniles (50 g body weight), were obtained from Piscimar fish farm (Andromeda Group, Burriana, Spain) and maintained at the animal facilities of the Faculty of Biology at the University of Barcelona (Spain). After 2 weeks acclimation period, fish were randomly distributed into three 200 L glass tanks (11 fish per tank and condition) under a 12 h light/12 h dark photoperiod, at room temperature (19 ± 1°C). The experiment was performed in January. Fish were daily fed *ad libitum* twice with a commercial diet (Skretting, Burgos, Spain). At the beginning of the trial, all three tanks started at the same temperature of 19°C and then, two of them went from 19 to 24°C or 28°C, with a rate of Δ1°C each day following the protocol of Hevrøy et al. (6) with a 250 W thermostatic heater (EHEIM, Deizisau, Germany). Once the water temperature required was achieved, fish were held for 3 more days and sampled on the day fourth. A schema of the experimental trial is shown in Figure 1. The temperature of the tanks was registered with a precision thermometer (Sera®) three times a day to ensure the corresponding temperature was maintained. Before sampling, fish were fasted for 24 h and then were anesthetized (MS-222 150 mg/L) and subsequently sacrificed by a blow to the head. Samples of white muscle and vertebral bone were collected and immediately frozen in liquid nitrogen and stored at −80°C until further analyses, and small fragments of bone were also used to perform the primary cultures just after sampling as explained in section Primary Culture of Bone-Derived Cells.

All animal handling procedures were approved by the Ethics and Animal Care Committee of the University of Barcelona, in accordance with the guidelines of the European Union Council (86/609/EU), and the Spanish and Catalan Government assigned principles and legislation (permit numbers DAAM 6759 and 9336 for the *in vitro* and *in vivo* experiments, respectively).

### Primary Culture of Bone-Derived Cells

Bone fragments of 10 fish per temperature condition were used per culture, each one considered an independent replicate, following the protocol of Capilla et al. (21). Briefly, the vertebras were removed, cleaned of all adherent tissues and washed twice in phosphate buffered saline with 1% antibiotic/antimycotic solution (A/A) prior to manually obtain with a scalpel small (<1 mm) fragments. After that, two digestions of 30 and 90 min, respectively, were done with 0.125% Type II collagenase in Hank's balanced salt solution at 18°C with gentle agitation. Next, the fragments were washed twice with Dulbecco's Modified Eagle Medium (DMEM) supplemented with 1% A/A solution and finally cultured in complete growth medium composed of DMEM supplemented with 10% fetal bovine serum and 1% A/A solution. Cells were seeded into 6 or 12-well plates and incubated at 23°C and 2.5% CO_2_. Medium was changed every 2 days. As indicated in the following sections, the fragments were removed from the plates at different days after seeding in order to perform the corresponding assays with the cells attached. To investigate whether temperature could affect the phenotype and differentiation of bone-derived cells in culture, pictures at days 8 and 15 of development were taken with a Canon EOS 1000D digital camera. All cell-culture reagents were purchased from Sigma–Aldrich (Tres Cantos, Spain) and all plastic items were obtained from Nunc (Labclinics, Barcelona, Spain).

### Viability Assay

The methylthiazolyldiphenyl-tetrazolium bromide (MTT) assay was used to evaluate cell viability as previously done in gilthead sea bream bone cells (21) and muscle cells (29). This method was selected since comparative studies of different viability assays regularly used revealed that it is the most sensitive one in terms of detecting cytotoxicity (30). Briefly, on days 8 and 15 cell samples of two duplicate wells of the 12 well-plates were incubated for 3 h in DMEM with a final concentration of 5 mg/mL of MTT (Sigma-Aldrich). Then, cells were washed with phosphate buffered saline and resuspended in 150 μL of dimethyl sulfoxide (DMSO) per well. The viability values were obtained from the absorbance measured at 570 nm in duplicate 96-wells, with correction at 650 nm, using a microplate reader (Tecan Infinite 200). Data from day 15 cells are presented as fold change relative to each corresponding day 8 of culture (*n* = 10).

### Mineralization Assay

Culture differentiation was evaluated according to mineralization of the ECM. The deposition of minerals was analyzed in day 20 cultured cells by alizarin red S (ARS) staining, following the protocol of Capilla et al. (21). Cells were fixed for 15 min with 10% formalin and stained with 2% ARS (pH 4.1–4.3) during 20 min. After washing excessive dye with water, quantification of the staining was done by means of acid extraction of the ARS stain with 10% acetic acid. The monolayer was then scrapped and transferred to a 1.5 ml tube. After vortex, the slurry was overlaid with mineral oil (Sigma–Aldrich), heated to 85°C for 10 min, cooled on ice and centrifuged at 16,000 g for 15 min. At this point, 10% ammonium hydroxide was added to the supernatant to neutralize the acid and finally, aliquots of the different samples were read at 405 nm in duplicate 96-wells, using a microplate reader (Tecan Infinite 200). Data are presented as optical density arbitrary units (*n* = 10).

### Gene Expression Analyses

#### RNA Extraction and cDNA Synthesis

Total RNA was extracted from ~100 mg of vertebral bone and white muscle tissues, or from cell samples of two duplicate wells of the 6 well-plates at day 15 with 1 mL of TRI Reagent Solution (Applied Biosystems, Alcobendas, Spain) following the manufacturer's instructions. Total concentration and purity were determined using a NanoDrop 2000 (Thermo Scientific, Alcobendas, Spain), and integrity of the different samples was confirmed in a 1% agarose gel (w/v) stained with SYBR-Safe DNA Gel Stain (Life Technologies, Alcobendas, Spain). Next, 1,000 ng of total RNA were treated with DNase I (Life Technologies) to remove all genomic DNA, and reverse transcribed with the Transcriptor First Strand cDNA Synthesis Kit (Roche, Sant Cugat del Valles, Spain). The cDNA obtained was stored at −20°C for real-time quantitative PCR analyses (qPCR).

#### Real-Time Quantitative PCR

The mRNA transcript levels of the target genes plus three reference genes were examined in a CFX384™ real-time system (Bio-Rad, El Prat de Llobregat, Spain). All the analyses were performed in triplicate wells using 384-well plates with 2.5 μL of iTaq Universal SYBR Green Supermix (Bio-Rad), 250 nM final concentration of forward and reverse primers (Table 1) and 1 μL of diluted cDNA for each sample, in a final volume of 5 μL. As described before (31, 32), reactions were performed with an initial activation step of 3 min at 95°C, 40 cycles of 10 s at 95°C and 30 s at 55–68°C (primer-dependent, see Table 1) followed by an amplicon dissociation analysis from 55 to 95°C at 0.5°C increase each 30 s. Before the analyses, a dilution curve with a pool of samples was run to confirm primer efficiency, specificity of the reaction, absence of primer-dimers, and to determine the appropriate cDNA dilution for each assay. Negative controls [no template control (NTC), no reverse transcriptase control (RTC) and MilliQ water (PCR)] were included and run in duplicate. The expression level of each target gene analyzed was calculated using the Pfaffl method (33), relative to the geometric mean of the two most stable reference genes determined for each tissue by the geNorm algorithm, both implemented in the Bio-Rad CFX manager 3.1. software.

**Table 1:** Primers used in the qPCR analyses: sequences, melting temperatures (Tm), and GenBank accession numbers.

**Gene**	**Primer sequences (5^**′**^-3^**′**^)**	**Tm, ^**°**^C**	**Accession number**
*rps18*	F: GGGTGTTGGCAGACGTTAC	60	AM490061.1
	R: CTTCTGCCTGTTGAGGAACCA		
*ef1a*	F: CTTCAACGCTCAGGTCATCAT	60	AF184170
	R: GCACAGCGAAACGACCAAGGGGA		
*rpl27a*	F: AAGAGGAACACAACTCACTGCCCCAC	68	AY188520
	R: GCTTGCCTTTGCCCAGAACTTTGTAG		
*hsp30*	F: GGTGACGGGAAAGAGA	60	GU60312
	R: CTGAGGAGGAGGTGCTGTTC		
*hsp90b*	F: TTCACGCATGGAAGAAGTTG	56	DQ012949
	R: GGTCCACCACACAACATGAA		
*pcna*	F: TGTTTGAGGCACGTCTGGTT	58	NM_131404.2
	R: TGGCTAGGTTTCTGTCGC		
*igf-1*	F: ACAGAATGTAGGGACGGAGCGAATGGAC	60	EF688016
	R: TTCGGACCATTGTTAGCCTCCTCTCTG		
*igf-2*	F: TGGGATCGTAGAGGAGTGTTGT	60	AY996778
	R: CTGTAGAGAGGTGGCCGACA		
*igfbp-1a*	F: AGTGCGAGTCCTCTCTGGAT	60	KM522771
	R: TCTCTTTAAGGGCACTCGGC		
*igfbp-2b*	F: CGGGCTGCTGCTGACATACG	60	AF377998
	R: GTCCCGTCGCACCTCATTTG		
*igfbp-4*	F: TCCACAAACCAGAGAAGCAA	68	F5T95CD02JMZ9K
	R: GGGTATGGGGATTGTGAAGA		
*igfbp-5b*	F: TTTCTCTCTCGGTGTGC	60	AM963285
	R: TCAAGTATCGGCTCCAG		
*ghr-1*	F: ACCTGTCAGCCACCACATGA	60	AF438176
	R: TCGTGCAGATCTGGGTCGTA		
*ghr-2*	F: GAGTGAACCCGGCCTGACAG	60	AY573601
	R: GCGGTGGTATCTGATTCATGGT		
*igf-1ra*	F: AGCATCAAAGACGAACTGG	55	KT156846
	R: CTCCTCGCTGTAGAAGAAGC		
*igf-1rb*	F: GCTAATGCGAATGTGTTGG	55	KT156847
	R: CGTCCTTTATGCTGCTGATG		
*runx2*	F: ACCCGTCCTACCTGAGTCC	60	JX232063
	R: AGAAGAACCTGGCAATCGTC		
*fib1a*	F: CGGTAATAACTACAGAATCGGTGAG	60	FG262933
	R: CGCATTTGAACTCGCCCTTG		
*bmp2*	F: GGAGAAGCAGCGTGGATTAAACACGAAT	65	AY500244
	R: GGCCTGCGCCTCAGTCCAAACATATT		
*bmp4*	F: CACGCCATTGTTCAGACACT	60	FJ436409
	R: GCCCTCCACTACCATTTCCT		
*mgp*	F: TGTGTAATTTATGTAGTTGTTCTGTGGCATCTCC	68	AY065652
	R: CGGGCGGATAGTGTGAAAATGGTTAGTG		
*on*	F: AGGAGGAGGTCATCGTGGAAGAGCC	68	AY239014
	R: GTGGTGGTTCAGGCAGGGATTCTCA		
*op*	F: AAAACCCAGGAGATAAACTCAAGACAACCCA	68	AY651247
	R: AGAACCGTGGCAAAGAGCAGAACGAA		
*ocn*	F: TCCGCAGTGGTGAGACAGAAG	56	AF048703
	R: CGGTCCGTAGTAGGCCGTGTAG		
*pax7*	F: ATGAACACTGTCGGCAACG	64	JN034418
	R: AGGCTGTCCACACTCTTGATG		
*myf5*	F: CTACGAGAGCAGGTGGAGAACT	64	JN034420
	R: TGTCTTATCGCCCAAAGTGTC		
*myod1*	F: TTTGAGGACCTGGACCC	60	AF478568.1
	R: CTTCTGCGTGGTGATGGA		
*myod2*	F: CACTACAGCGGGGATTCAGAC	60	AF478569
	R: CGTTTGCTTCTCCTGGACTC		
*myogenin*	F: CAGAGGCTGCCCAAGGTGGAG	68	EF462191
	R: CAGGTGCTGCCCGAACTGGGCTCG		
*mrf4*	F: CATCCCACAGCTTTAAAGGCA	60	JN034421
	R: GAGGACGCCGAAGATTCACT		
*mstn1*	F: GTACGACGTGCTGGGAGACG	60	AF258448.1
	R: CGTACGATTCGATTCGCTTG		
*mstn2*	F: ACCTGGTGAACAAAGCCAAC	60	AY046314
	R: TGCGGTTGAAGTAGAGCATG		
*cd36*	F: GTCGTGGCTCAAGTCTTCCA	60	-
	R: TTTCCCGTGGCCTGTATTCC		
*fatp1*	F: CAACAGAGGTGGAGGGCATT	60	-
	R: GGGGAGATACGCAGGAACAC		
*fabp11*	F: CATTTGAGGAGACCACCGCT	60	-
	R: ACTTGAGTTTGGTGGTACGCT		
*hsl*	F: GCTTTGCTTCAGTTTACCACCATTTC	60	EU254478
	R: GATGTAGCGACCCTTCTGGATGATGTG		
*lipa*	F: TACTACATCGGACACTCTCAAGGAAC	60	JQ308831
	R: GTGGAGAACGCTATGAATGCTATCG		
*lpl-lk*	F: CAGAGATGGAGCCGTCACTCAC	60	JQ390609
	R: TCTGTCACCAGCAGGAACGAATG		
*lmf1*	F: CGGCTGGACTGGCTCATGT	60	JX975718
	R: CTCACTCTGCTCGTAGGTCTGGAA		
*cpt1a*	F: GTGCCTTCGTTCGTTCCATGATC	60	JQ308822
	R: TGATGCTTTATCTGCTGCCTGTTTG		
*cpt1b*	F: CCACCAGCCAGACTCCACAG	60	DQ866821
	R: CACCACCAGCACCCACATATTTAG		
*hadh*	F: GAACCTCAGCAACAAGCCAAGAG	60	JQ308829
	R: CTAAGAGGCGGTTGACAATGAATCC		
*ucp2*	F: CGGCGGCGTCCTCAGTTG	60	JQ859959
	R: AAGCAAGTGGTCCCTCTTTGGTCAT		

*F, forward; R, reverse. rps18, ribosomal protein s18; ef1a, elongation factor 1 alpha; rpl27a, ribosomal protein l27a; hsp30, heat shock protein 30; hsp90b, heat shock protein 90b; pcna, proliferating cell nuclear antigen; igf-1, insulin-like growth factor 1; igf-2, insulin-like growth factor 2; igfbp-1a, insulin-like growth factor binding protein 1a; igfbp-2b, insulin-like growth factor binding protein 2b; igfbp-4, insulin-like growth factor binding protein 4; igfbp-5b, insulin-like growth factor binding protein 5b; ghr-1, growth hormone receptor 1; ghr-2, growth hormone receptor 2; igf-1ra, insulin-like growth factor 1 receptor a; igf-1rb, insulin-like growth factor 1 receptor b; runx2, runt-related transcription factor 2; fib1a, fibronectin subunit 1a; bmp2, bone morphogenetic protein 2; bmp4, bone morphogenetic protein 4; mgp, matrix gla protein; on, osteonectin; op, osteopontin; ocn, osteocalcin; pax7, paired box 7; myf5, myogenic factor 5; myod1, myogenic differentiation 1; myod2, myogenic differentiation 2; myogenin, myogenin; mrf4, myogenic regulatory factor 4; mstn1, myostatin 1; mstn2, myostatin 2; cd36, cluster of differentiation 36; fatp1, fatty acid transport protein 1; fabp11, fatty acid binding protein 11; hsl, hormone sensitive lipase; lipa, lipase a; lpl-lk, lipoprotein lipase-like; lmf1, lipase maturation factor 1; cpt1a, carnitine palmitoyltransferase 1a; cpt1b, carnitine palmitoyltransferase 1b; hadh, hydroxyacil-CoA dehydrogenase; ucp2, uncoupling protein 2*.

### Statistical Analysis

Data were analyzed using IBM SPSS Statistics v. 22 (IBM, Armonk, USA) and are presented as Mean ± SEM. Data normality and homoscedasticity were tested among temperature groups using Shapiro–Wilk and Levene's tests, respectively. Statistical significance was assessed by one-way analysis of variance (one-way ANOVA) followed by Tukey's *post-hoc* test. When homoscedasticity was not observed Dunnett T3 test was applied. Statistical differences were considered significant for all analysis when *p* < 0.05.

## Results

### HSPs and Proliferation Marker Genes Expression in Bone and White Muscle Tissues

**Figure 2:**
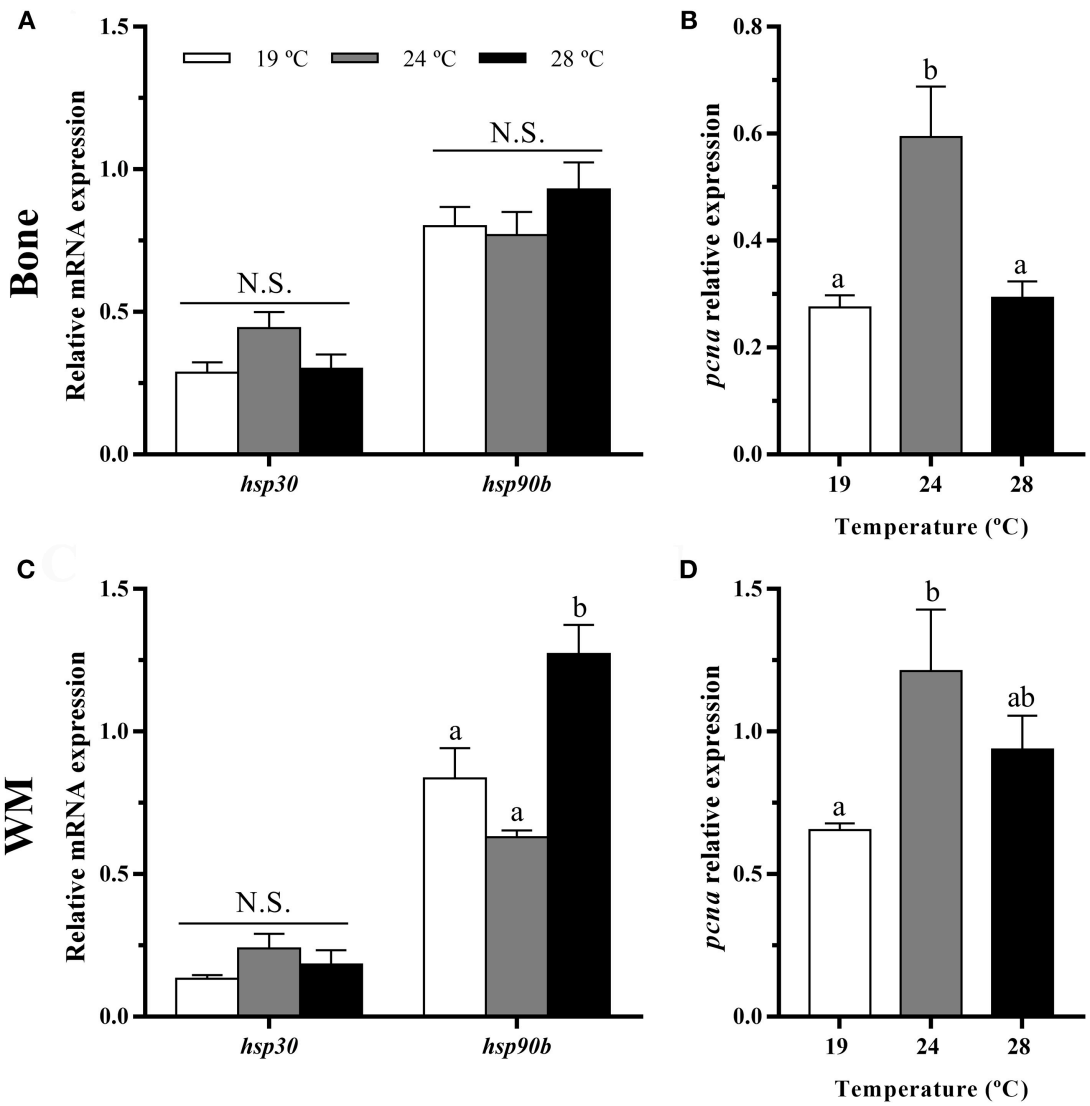
*In vivo* effects of temperature over the gene expression of heat shock proteins and a proliferation marker in gilthead sea bream in **(A,B)** bone and **(C,D)** white muscle (WM). Relative mRNA expression normalized to *ef1a* and *rps18* of **(A,C)** *hsp30* and *hsp90b* and **(B,D)** *pcna*. Data are shown as Mean + SEM (*n* = 8). Significant differences among fish held at different temperatures were determined by one-way ANOVA and are indicated by different letters (*p* < 0.05). N.S., non-significant.

In bone, heat shock proteins, *hsp30* and *hsp90b* mRNA levels were similar among the three groups (Figure 2A), while the temperature of 24°C caused a significant increase in the gene expression of the proliferating cell nuclear antigen (*pcna*) respect to the other groups (Figure 2B).

In white muscle, *hsp30* remained also unaltered, but *hsp90b* gene expression was highest at 28°C (Figure 2C). Concerning *pcna*, a significant up-regulation at 24°C compared with the 19°C condition as in bone was observed (Figure 2D).

### GH-IGFs Axis-, Osteogenic-, and Myogenic-Related Genes Expression in Bone and White Muscle Tissues

**Figure 3:**
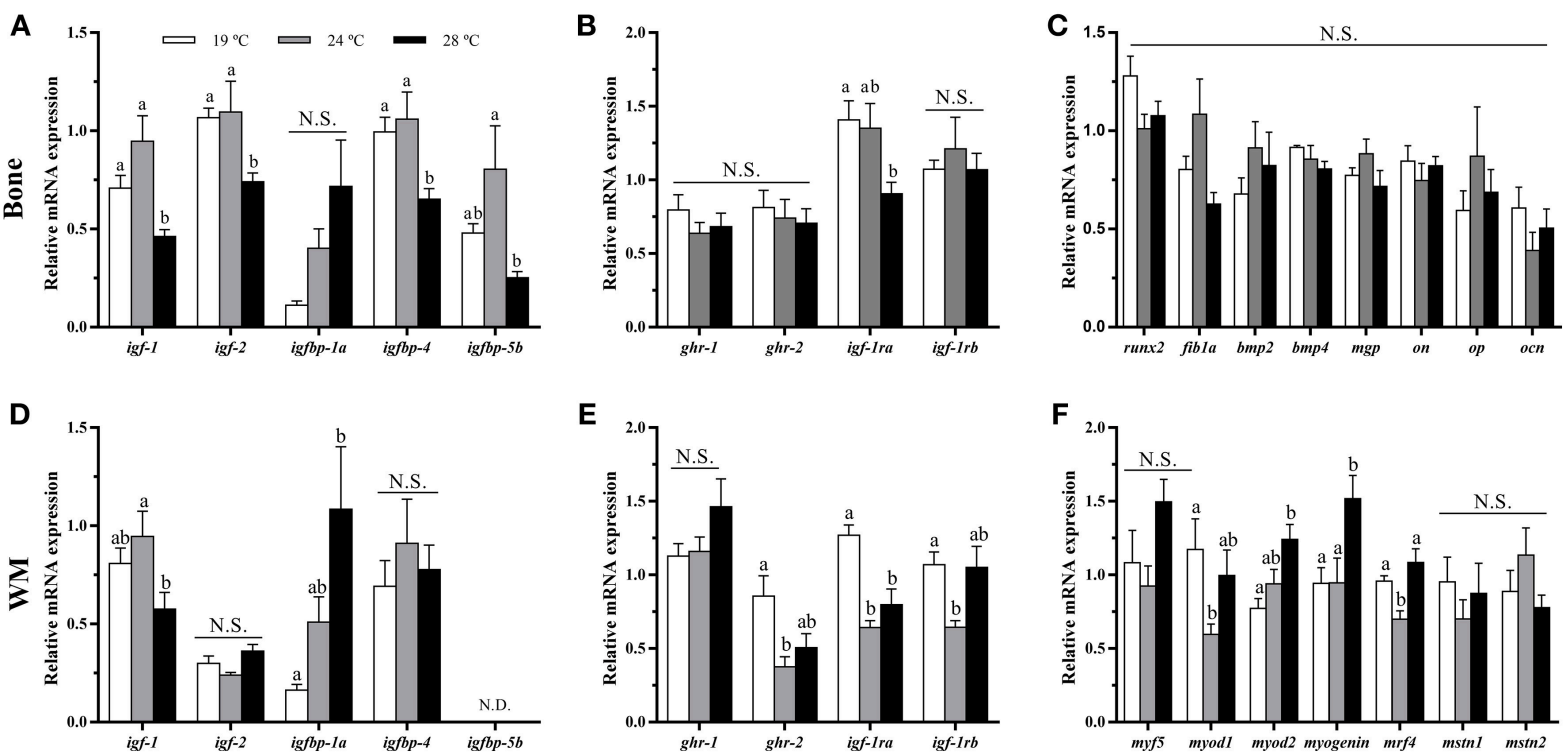
*In vivo* effects of temperature over the expression of GH-IGFs axis-, osteogenic-, and myogenic-related genes in **(A–C)** bone and **(D–F)** white muscle (WM). Relative mRNA expression normalized to *ef1a* and *rps18* of **(A,D)** *igf-1, igf-2*, and *igf* binding proteins (*1a, 4*, and *5a*), **(B,E)** *gh* and *igf-1* receptors, **(C)** *runx2, fib1a, bmp2, bmp4, mgp, on, op*, and *ocn*, and **(F)** *myf5, myod1, myod2, myogenin, mrf4, mstn1*, and *mstn2*. Data are shown as Mean + SEM (*n* = 8). Significant differences among fish held at different temperatures were determined by one-way ANOVA and are indicated by different letters (*p* < 0.05). N.S., non-significant; N.D., non-detectable.

In bone, the mRNA levels of *igf-1, igf-2, igfbp-4, igfbp-5b*, and *igf-1ra* were significantly down-regulated at 28°C compared with the 19°C reared fish and, in most cases, compared to the 24°C condition as well (Figures 3A,B). Contrarily, *igfbp-1a, ghr-1, ghr-2*, and *igf-1rb* did not show differences among groups, although the former presented a tendency to gradually increase along with temperature. Concerning the osteogenic genes analyzed, none of them showed significant differences in response to temperature treatment under the experimental *in vivo* conditions tested (Figure 3C).

In white muscle, *igf-1* mRNA levels were lower at 28°C, although differences were only significant compared to 24°C-exposed fish (Figure 3D). Moreover, *igfbp-1a* showed the same pattern as observed in bone tissue, significantly increasing its expression along with temperature. *igf-2* and *igfbp-4* did not revealed differences among groups, and *igfbp-5b* was not detectable in this tissue. Furthermore, the levels of expression of *gh* and *igf-1 receptors* were unaltered for *ghr-1* compared with the group of 19°C but were significantly lower for *ghr-2* and *igf-1rb* at 24°C and for *igf-1ra* at 24 and 28°C (Figure 3E). With regards to the myogenic-related genes, the expression of *myod1* and *mrf4* was significantly lower in the 24°C-held fish compared to the other two groups, while *myod2* and *myogenin* mRNA levels were significantly higher in the fish kept at 28°C, and neither muscle growth inhibitor, *mstn1* nor *mstn2*, were affected by the rearing temperature (Figure 3F).

In addition, the expression of *igfbp-2b* was analyzed in both tissues, although none of them showed detectable levels.

### Lipid Metabolism-Related Genes Expression in White Muscle Tissue

**Figure 4:**
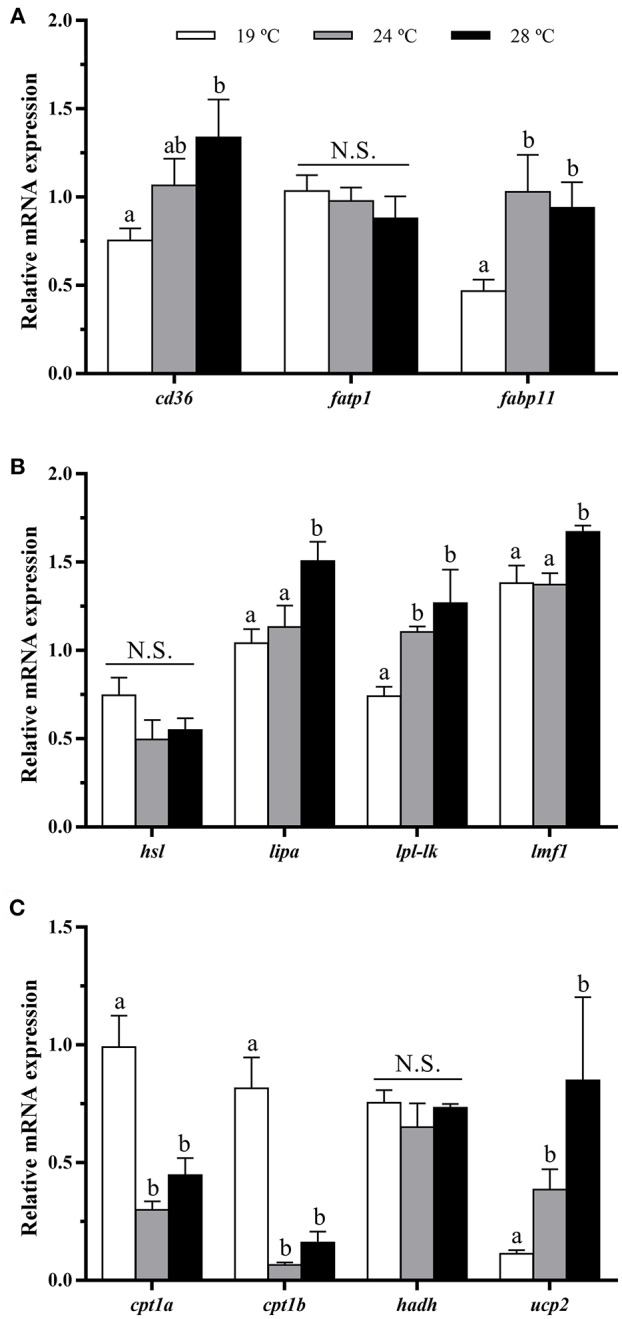
*In vivo* effects of temperature over the expression of lipid metabolism-related genes in white muscle. Relative mRNA expression normalized to *rps18* and *rpl27a* of **(A)** fatty acid transporters *cd36, fatp1, fabp11*, **(B)** lipases *hsl, lipa* and *lp-lk, lmf1*, and **(C)** β-oxidation markers *cpt1a, cpt1b, hadh*, and *ucp2*. Data are shown as Mean + SEM (*n* = 8). Significant differences among fish held at different temperatures were determined by one-way ANOVA and are indicated by different letters (*p* < 0.05). N.S., non-significant.

The fatty acid transporter *cd36* was significantly up-regulated with the temperature rise, showing the fish at 28°C the highest mRNA levels. The expression of *fatp1* remained unaltered, but *fabp11* was significantly enhanced at 24 and 28°C compared with the 19°C group (Figure 4A). Concerning lipases, although *hsl* mRNA levels were not different among groups, *lipa* and *lpl-lk* transcript levels were significantly increased in 28°C, or 24 and 28°C-exposed fish, respectively, compared with the 19°C group. In parallel to this, significant up-regulation of the lipase maturation factor (*lmf1*, an essential gene for the folding and assembly of LPL) was detected in the 28°C-reared fish (Figure 4B). Regarding β-oxidation markers, the gene expression of mitochondrial carnitine palmitoyltransferases (*cpt1a* and *cpt1b*) was significantly lower in juveniles maintained at 24 and 28°C when compared with fish reared at 19°C, while hydroxyacyl-CoA dehydrogenase *hadh* expression did not show significant differences among groups. Contrarily, the mitochondrial uncoupling protein *ucp2* showed significantly higher mRNA levels with increased temperature (Figure 4C).

### Characterization of the Bone-Derived Cells Culture

**Figure 5:**
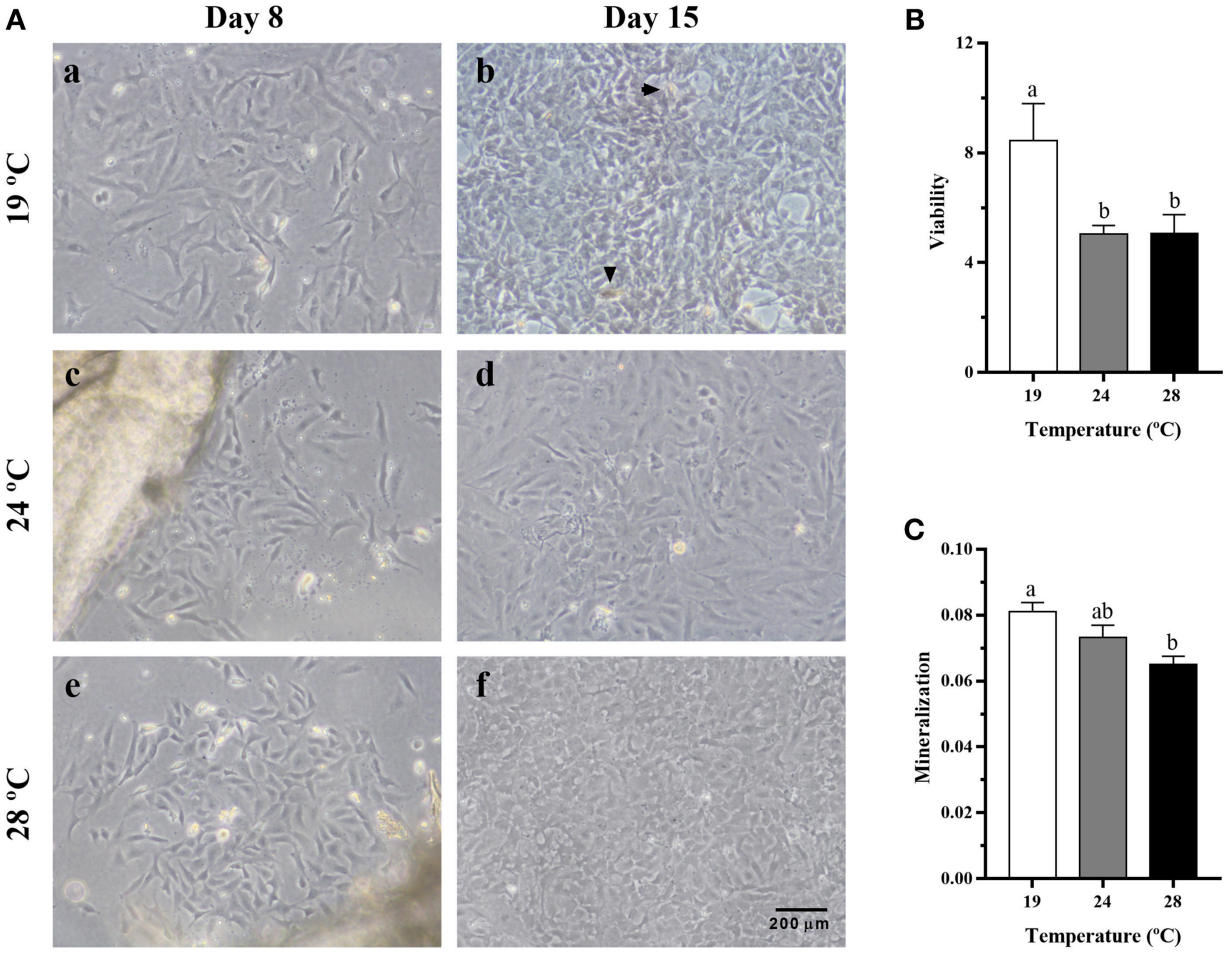
**(A)** Representative images of cells derived from vertebrae bone of gilthead sea bream reared at different temperatures, at **(a,c,e)** day 8 and **(b,d,f)** day 15 of culture development. Magnification, 10x. Arrowheads indicate the presence of mineral nodules. **(B)** Quantification of viability in gilthead sea bream cultured bone-derived cells using an MTT assay presented as fold change of day 15 relative to day 8 of culture. **(C)** Quantification of mineralization in gilthead sea bream cultured bone-derived cells at day 20 determined by ARS staining. Data are shown as Mean + SEM (*n* = 10). Different letters among temperature groups indicate significant differences, calculated by one-way ANOVA (*p* < 0.05).

The effects of temperature during the differentiation of gilthead sea bream bone-derived cells are presented in Figure 5A. Morphologically, in all three groups, the cells showed at day 8 a spindle-like phenotype that changed to a polygonal one at day 15 as the cells differentiated spontaneously into osteoblasts. Moreover, deposits of minerals started to accumulate in the ECM, although mineral nodules were apparently in greater proportion in cells derived from the 19°C-reared fish compared to the other two groups. Besides, as shown in Figure 5B, significantly lower viability values were found in cells coming from fish reared at 24 and 28°C, compared with those at 19°C; and the same was observed concerning the deposition of minerals in the ECM (Figure 5C), which agreed with the visual observation.

### HSPs and Proliferation Marker Genes Expression in Bone-Derived Cells

**Figure 6:**
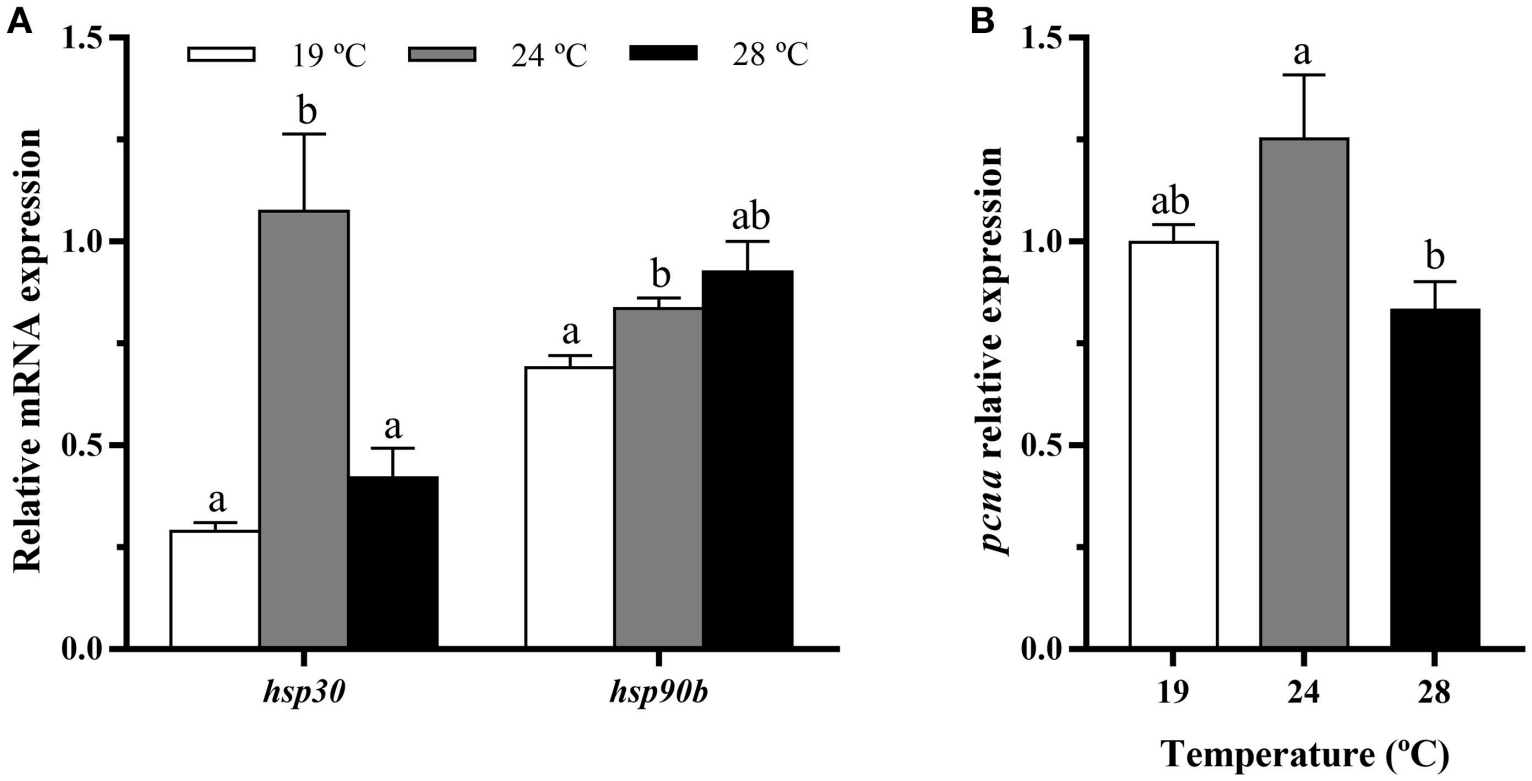
*In vitro* effects of fish rearing temperature over the gene expression of heat shock proteins and a proliferation marker in bone-derived cells at day 15 of culture development. Relative mRNA expression normalized to *ef1a* and *rps18* of **(A)** *hsp30* and *hsp90* and **(B)** *pcna*. Data are shown as Mean + SEM (*n* = 6–7). Significant differences among temperatures groups gene were determined by one-way ANOVA and are indicated by different letters (*p* < 0.05).

Exposure of gilthead sea bream juveniles to 24°C of temperature significantly increased the expression of *hsp30* and *hsp90b* transcript levels in bone-derived cultured cells compared to those coming from the other fish (Figure 6A). In addition, a significant down-regulation of the proliferation marker *pcna* gene expression was detected in the 28°C-cells compared with those obtained from 24°C-held fish (Figure 6B).

### GH-IGFs Axis- and Osteogenic-Related Genes Expression in Bone-Derived Cells

**Figure 7:**
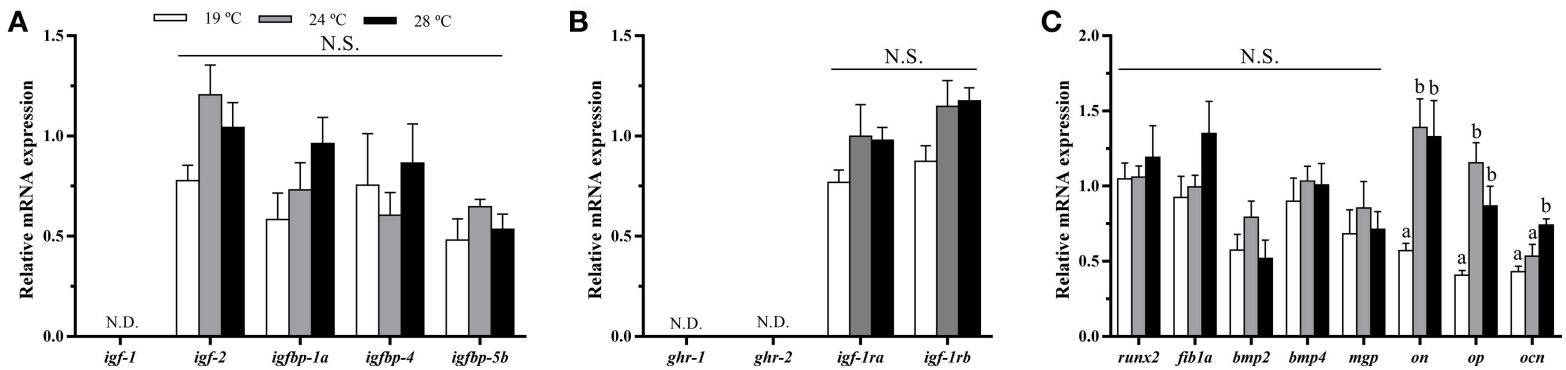
*In vitro* effects of fish rearing temperature over the expression of GH-IGFs axis- and osteogenic-related genes in bone-derived cells at day 15 of culture development. Relative mRNA expression normalized to *ef1a and rps18* of **(A)** *igf-1, igf-2* and *igf* binding proteins (*1a, 4*, and *5a*), **(B)** *gh* and *igf-1* receptors and **(C)** *runx2, fib1a, bmp2, bmp4, mgp, on, op*, and *ocn*. Data are shown as Mean + SEM (*n* = 6–7). Significant differences among temperatures groups were determined by one-way ANOVA and are indicated by different letters (*p* < 0.05). N.S., non-significant; N.D., non-detectable.

The analysis of GH-IGFs system-related genes expression in cultured bone-derived cells revealed no differences among groups (Figures 7A,B). Regarding the expression of osteogenic genes, significant differences were neither observed for most of them but significant up-regulation of *on, op*, and *ocn* gene expression was found in cells coming from gilthead sea bream maintained at elevated temperatures, compared with those cells from 19°C-reared fish (Figure 7C). To corroborate the determination of the bone-derived cultured cells toward the osteogenic lineage, the gene expression of *pax7*, one of the main transcription factors shaping the fate of MSCs into the muscular lineage, was analyzed, resulting undetectable.

## Discussion

As existing literature reports, an increment of water temperature has been proved to be a valid approach to evaluate impact of global climate change on physiological responses in various fish species (6, 15, 25, 34, 35). Besides, we have recently described in gilthead sea bream temperature-dependent differential expression of genes involved in osteogenesis, indicating a modulation of bone formation caused by this abiotic factor (20). In the current study, the aim was to characterize the effects of increased temperature in gilthead sea bream juveniles' musculoskeletal growth, muscle lipid metabolism and, in the *in vitro* development of primary cultured bone-derived cells to test the hypothesis that global climate change modulates the expression of key genes locally in bone and muscle, which might increase the occurrence of skeletal anomalies in this species.

### Effects of Temperature on Cell Culture Development

The bone-derived cultured cells from gilthead sea bream vertebrae gave an initial homogenous population of cells. At the first stages, cells were mostly triangular as previously reported for this species (21) and mammalian bone marrow stem cells (36) and, up to day 8 there were no differences in morphology among the three temperature groups. At day 15, cell differentiation became more evident, with the change into a polygonal shape, characteristic of the osteoblast phenotype (36). These changes followed the profile also reported for gilthead sea bream either in primary cultured cells (21) or the osteoblast-like VSa16 cell line (37). This change in morphology together with the absent levels of *pax7* and the elevated expression of osteogenic-genes, confirmed that all three cultures performed in the present study were determined toward the osteogenic lineage. Nevertheless, the increase in temperature appeared to lead toward a disrupted, or at least retarded, osteogenic process, since a decrease in cell viability and mineralization was observed in bone cells derived from 24 to 28°C-held fish compared with the 19°C group. Reduced mineralization caused by a high-temperature treatment was also found by Ytteborg et al. (38) in Atlantic salmon vertebral tissue, supporting this hypothesis.

### Effects of Temperature on *hsps* and *pcna* Gene Expression *in vivo* and *in vitro*

Although the gene expression of *hsp90b* was increased *in vivo* only in the muscle tissue under the highest temperature tested, the *in vitro* experiment showed direct evidence of water temperature up-regulating *hsp30* and *hsp90b*; thus, supporting the stressful condition induced to the animals. A similar response was reported for both genes in the same gilthead sea bream *in vitro* model when similar temperature changes were applied directly into the cells (20). Previous studies in larvae of sole and grass carp (*Ctenopharyngodon idella*) also described an increased expression of *hsp90* in response to a temperature rise (39, 40), as a protective mechanism against thermal stress. In fact, an increase in *hsp30* mRNA levels was observed only after 3 h of an *in vivo* temperature increase in rainbow trout (*Oncorhynchus mykiss*) (41). Similar activations of chaperones involved in protein folding have been reported in muscle tissue of gilthead sea bream (42) and rainbow trout (43) facing another stressful situation such as fasting.

The proliferation marker *pcna* showed the same pattern of expression *in vivo* in bone and muscle than *in vitro* in bone-cultured cells, with increased mRNA levels in 24°C-reared fish respect to the other groups. Thus, despite the cells showed reduced viability with the increase in temperature, this up-regulation of *pcna* could be considered a compensatory response, attempting these cells to recover from their initial heat stress-related situation. Accordingly, that could be considered a compensatory growth mechanism *in vivo*. Thus, it appears that 24°C would be the limiting temperature to properly grow gilthead sea bream, being the condition of 28°C fairly challenging. This is in agreement with the range of adequate rearing temperatures reported for this species (16–22°C) not causing significant harmful health effects and/or inducing skeletal malformations (14). Similarly, a previous study carried out in human osteosarcoma cells revealed that an increase in temperature outside optimum has a pronounced inhibitory effect on proliferation rate (44).

### Effects of Temperature on GH/IGFs Axis-Related Genes Expression *in vivo* and *in vitro*

The expression of *igf-1* was significantly decreased in both bone and muscle tissues of 28°C-held fish when compared with fish reared at 24 or 19°C, while its expression was not detectable in cultured osteoblasts. IGF-1 plays an important role inducing not only muscle differentiation and hypertrophy, but also bone matrix production (16, 45). The down-regulating effect of high temperature on *igf-1* observed in gilthead sea bream has been also reported in muscle of different fish species (i.e., Atlantic salmon and southern flounder), as well as, in IGF-1 plasma levels (6, 46, 47), suggesting restricted growth. Notwithstanding, similar studies in rainbow trout showed contrarily, an increase in plasma GH and IGF-1 levels with high temperature (7, 8), overall suggesting that the response to temperature increase of the major growth factors in fish could be species-specific. Regarding *igf-2*, changes were not observed among groups in muscle and cultured bone cells, although the same response as *igf-1* was observed in the bone *in vivo*, indicating that this tissue appears to be more sensitive to changes in temperature. This data is in agreement with that observed previously in unresponsive rainbow trout muscle, both at mRNA and plasma levels (8); however, in Atlantic salmon, Hevrøy et al. (6) found that *igf-2* mRNA levels were significantly down-regulated in muscle and liver after 45 days of exposition to warm temperature but not after only 15 days. Thus, it cannot be excluded that a prolonged trial time could have affected the expression of this gene in the present study as well.

Concerning the GH and IGF-1 receptors, juveniles held at 24 and 28°C presented in muscle significantly lower levels of expression of *igf-1ra* than at 19°C; those reared at 24°C also had decreased *igf-1rb* and *ghr-2*, and in bone those at 28°C also showed reduced *igf-1ra* mRNA levels. Wargelius et al. (16) revealed that an increase in the gene expression of *igf-1ra* relates with an increase in bone density in Atlantic salmon. Thus, the decrease in *igf-1ra* expression observed in gilthead sea bream could lead to reduced mineralization in the long-term caused by the high rearing temperature, which would be in agreement with that observed by Ytteborg et al. (48) in the former species. Therefore, in this context, the results of the present study suggest that the GH/IGFs axis is influenced in gilthead sea bream by elevated temperature to locally decrease the expression of *ghrs, igfs*, and *igf-1rs* in bone and muscle in order to delay musculoskeletal growth. Interestingly, although differences exist between the *in vivo* and *in vitro* data, which could be due to modulation of the gene expression by systemic factors in the whole animal, the results obtained in the bone-derived cultured cells reflect this impaired situation as well.

With regards to the IGFBPs of major local action in the musculoskeletal tissues, the same increasing pattern in expression with temperature was observed concerning *igfbp-1a* in white muscle and bone, as previously found in Atlantic salmon muscle (6). Previous studies in zebrafish (*Danio rerio*) revealed that elevated expression of *igfbp-1a* limits cellular actions of IGF-1, being an important growth and developmental inhibitor (49). Moreover, this binding protein has been associated with stressful or negative conditions, since a strong relation with elevated serum cortisol levels has been reported (50, 51). In this context, the gradual increase of *igfbp-1a* along with temperature observed in this study, suggests impaired growth conditions in agreement with the reduced expression of *igfs* observed at 28°C. Furthermore, *igfbp-4* and *igfbp-5b* mRNA levels were also significantly decreased in bone tissue of 28°C-held fish, although remained unaltered in muscle and bone cells. In previous studies, these binding proteins have been reported as positive regulators of IGF-1 actions, with *igfbp-5b* promoting bone differentiation (45); and its mRNA levels being highly correlated with *igf-1* and *igf-2* in muscle (10, 32, 52, 53). Therefore, altogether, the down-regulation of *igfbp-4* and *igfbp-5b* in bone tissue and the increase of *igfbp-1a* in muscle of gilthead sea bream maintained at 28°C, indicates that such high temperature is an unfavorable condition, leading to reduced musculoskeletal growth and differentiation in this species.

### Effects of Temperature on Osteogenic- and Myogenic-Related Genes Expression *in vivo* and *in vitro*

In the present study, although none of the osteogenic-related genes analyzed showed differences *in vivo*, increasing water temperature was sufficient to induce an up-regulating response on some genes involved in the mineralization of the ECM (*on, op*, and *ocn*) in the cultured bone-derived cells, suggesting that the thermal history can influence the developmental plasticity of the osteogenic process *in vitro*. Interestingly, the ECM glycoprotein ON has been reported as a heat shock protein having chaperone-like properties to prevent collagen denaturation (54, 55). Therefore, as it was observed in rainbow trout by Currie et al. (41) and in a previous study by our group using the same cellular system (20), the increase in *on* expression caused by changes in temperature could potentially represent an initial response of bone cells to stressful conditions. According to this, the increase in *on* mRNA levels may also suggest a negative effect for ECM production and mineralization, which agrees with the reduced number of deposited minerals in the ECM along with temperature in the bone-cultured cells of the present study. Moreover, the elevated expression of *op*, a well-known inhibitor of matrix mineral deposition (56) supports this improper mineralization of the bone when fish are maintained at high temperatures. However, the ability of OP to regulate this process depends on its state of phosphorylation (57), thus, further analyses should be done to confirm this hypothesis.

Concerning *in vivo* studies, after a long-term high-temperature treatment in Atlantic salmon, Ytteborg et al. (38) reported that the mRNA levels of *runx2* (the key transcription factor of osteogenesis), decreased when fish reached 15 g but not at 2 g of body weight; whereas other non-collagenic ECM molecules such as *ocn, on*, or *col1a1* were down-regulated already at the 2 g stage. These authors proposed that these results might suggest a defect in the late maturation of osteoblasts, which agrees with the lower mineral density and shorter length-height proportion observed in the vertebrae of these animals (38). It also agrees with the subsequent significantly increased incidence of malformations found in the fish reared at high temperature at body weights of 15 and 60 g. Moreover, the same authors observed, in an *in vitro* study with precursor muscle cells differentiated into osteoblasts and cultured at an elevated temperature, a reduced expression of *ocn* and *col1a1* (48). In gilthead sea bream, expression of osteogenic genes was modified by temperature in both embryo and larval stages, but in the juveniles, differences were only observed after producing a temperature challenge (20). In the same study, lower transcript levels of most of the osteogenic genes analyzed in cultured osteoblasts in response to a long-term treatment of increased temperature were reported. Overall, these data suggest that at a transcriptional level, the deleterious effects of temperature on bone development could depend on the time of exposure. Therefore, it cannot be discarded that a prolonged temperature treatment could have also affected the expression of osteogenic genes in gilthead sea bream *in vivo*, pointing out then that osteoblast differentiation and bone ECM mineralization could be impaired.

The coordinated expression of MRFs to properly control muscle development can be modulated by temperature in teleost fish, thus affecting muscle growth (42, 58). In the present study, *myod1* transcript levels were reduced in the fish maintained at 24°C, while *myogenin* and *mrf4* expression was highest in the 28°C-reared fish, suggesting potentially slackened cell proliferation but enhanced myocyte differentiation with the increase in water temperature, overall uncoupling the myogenic process. In this framework, with high rearing temperatures, gilthead sea bream musculoskeletal growth would not be under harmonic conditions, which could be leading in the long-term to increased prevalence of bone deformities.

### Effects of Temperature on Muscle Lipid Metabolism-Related Genes Expression *in vivo*

The increase of temperature, up-regulated in the present work the muscle expression of the fatty acid transporter and binding protein *cd36* and *fabp11*, respectively, suggesting elevated fatty acid uptake and intracellular transport, upon high-temperature conditions. These data are in accordance with a recent study by Zoladz et al. (59), which reported enhanced protein expression of CD36 in rat skeletal muscle under hyperthermia. Nevertheless, the function of FABP11, which is probably an isoform restricted to fishes, is not completely known (60). With regards to the endothelial enzymes with a triglycerides lipase activity, gilthead sea bream juveniles held at 28°C presented significantly higher mRNA levels of *lipa* and *lpl-lk*, an exclusive fish lineage isoform of LPL (59). Regulation of the latter enzyme is far from being established yet, although it is known that in skeletal muscle of gilthead sea bream, changes in LPL-like are correlated with LMF1 (61), an endoplasmic reticulum membrane protein involved in the post-translational folding and assembly of LPL, among other proteins (62). This agrees with the results of this study, where *lmf1* mRNA levels were also increased as temperature was raised. Contrarily, the increase of temperature did not induce significant changes in *hsl* gene expression among groups, suggesting that fatty acids are mostly being uptaken by the muscle from circulating triglycerides, or non-esterified fatty acids provided by adipose tissue. Recent studies in Atlantic salmon and catfish (*Pelteobagrus vachellii*) demonstrated a reduction in hepatic triglycerides and relative viscera weight during exposure to elevated temperatures (6, 63), which illustrated that lipid metabolism may have increased, accelerating utilization of lipids as an energy source in peripheral tissues. In accordance with this, in rainbow trout exposed to high temperatures, endogenous lipid stores remained the most important energy source contributing up to 55% of total demand (64).

Concerning β-oxidation, an association between water temperature and fatty acid catabolism has been shown, but with inconsistent results in the literature. A recent study in Atlantic salmon showed an increase of β-oxidation in white muscle with increased temperature (25), while changes were not observed by Hevrøy et al. (47), and another study in rainbow trout reported increased capacities for oxidizing lipids at cold temperatures (65). In any case, it has been generally accepted that liver and red muscle, but not white muscle, are the most important tissues involved in fatty acid catabolism in fish; thus, an increase in water temperature has been shown to induce increased β-oxidation primarily in those tissues (66, 67). Other study in salmon found that elevation of water temperature was responsible for reduced β-oxidation in liver (47). These last data would be in agreement with the present study, where *cpt1a* and *cpt1b* were down-regulated at 24 and 28°C compared with the low-temperature group, considering the flux of β-oxidation is primarily determined by CPT1, which allows long chain fatty acids to enter into the mitochondria (25).

Notwithstanding, temperature can also affect mitochondrial uncoupling. The increase of temperature from 19 to 28°C led in gilthead sea bream muscle to the up-regulation of the uncoupling protein *ucp2*, indicating a higher proton leak, which is in agreement with previous studies in skeletal muscle in mammals (68, 69). UCPs are known to be activated not only by free fatty acids, but also by reactive oxygen species (70, 71). According to this, *ucp2* may play a role as a mechanism for attenuating the possible increase in reactive oxygen species associated with elevated temperature; therefore, overall indicating a less efficient use of fatty acids to obtain energy in this species, upon these environmental conditions.

## Conclusions

To sum up, the present work reports that an increase in water rearing temperature from 19 to 24 and specially 28°C causes in gilthead sea bream juveniles unfavorable growth conditions for the musculoskeletal system due to reduced gene expression of members of the GH/IGFs system and specific MRFs. In white muscle as well, as energetic demand is increased along with temperature, the uptake of fatty acids is enhanced, although apparently, their use as an energy source is less efficient. In addition, the high temperatures applied *in vivo*, induced changes *in vitro* in the expression of several key osteogenic genes, suggesting reduced osteoblasts development and matrix production, consistent with the decrease observed in the deposition of minerals. Overall, the present study provides new insights into the possible impact of global climate change in this important Mediterranean species, demonstrating that temperature is a key environmental factor whose increase can lead to unbalanced muscle and bone growth, which should be considered to take preventive measures to reduce production losses and guarantee the sustainability and success of aquaculture.

## Author Contributions

MR-C and EC conceived and designed the experiments. SB-P, NR-H, and EV performed the experiments. JG, IN, MR-C, and EC contributed reagents and analysis tools. SB-P, NR-H, EV, JG, IN, MR-C, and EC drafted and critically reviewed the paper.

### Conflict of Interest Statement

The authors declare that the research was conducted in the absence of any commercial or financial relationships that could be construed as a potential conflict of interest.
